# Surface passivation engineering for stable optoelectronic devices via hydroxyl-free ZnMgO nanoparticles

**DOI:** 10.1186/s40580-025-00493-2

**Published:** 2025-06-09

**Authors:** Seongkeun Oh, Jaehwi Choi, Junhyeok Park, Young Kyun Choi, Taesung Park, Awais Ali, Junhyuk Ahn, Jiwan Kim, Soong Ju Oh

**Affiliations:** 1 Department of Materials Science and Engineering, Korea University, Seoul, 02841 Republic of Korea; 2 Department of Advanced Materials Engineering, Kyonggi University, Suwon-si, 16227 Republic of Korea

**Keywords:** Quantum dot, Surface engineering, Optoelectronic devices, Hydroxyl-free, Alcohol treatment

## Abstract

ZnMgO nanoparticles (ZMO NPs) are widely used as electron transport layers in optoelectronic devices such as light-emitting diodes (LEDs) and photodiodes (PDs) primarily because of their facile synthesis and excellent electron transport properties. However, the surface hydroxyl groups (‒OH) on the ZMO NPs introduce charge traps, inhibit electron transport, and reduce device stability, particularly under ambient humidity and oxygen. Therefore, in this study, an alcohol treatment (AT) method was developed to remove surface ‒OH via proton transfer to effectively reduce trap states and dipole moments and enhance surface passivation. Quantum-dot-based LEDs and PDs fabricated using the AT-based ZMO NPs exhibited improved current density, luminance, and external quantum efficiency compared to the untreated devices. Notably, the methanol-treated devices achieved an operational lifetime of approximately 28 h under ambient conditions, representing a substantial advancement in device stability and performance. The AT approach is a simple and effective strategy for optimizing the ZMO NPs for next-generation optoelectronic applications.

**Supplementary Information:**

The online version contains supplementary material available at 10.1186/s40580-025-00493-2.

## Graphical Abstract

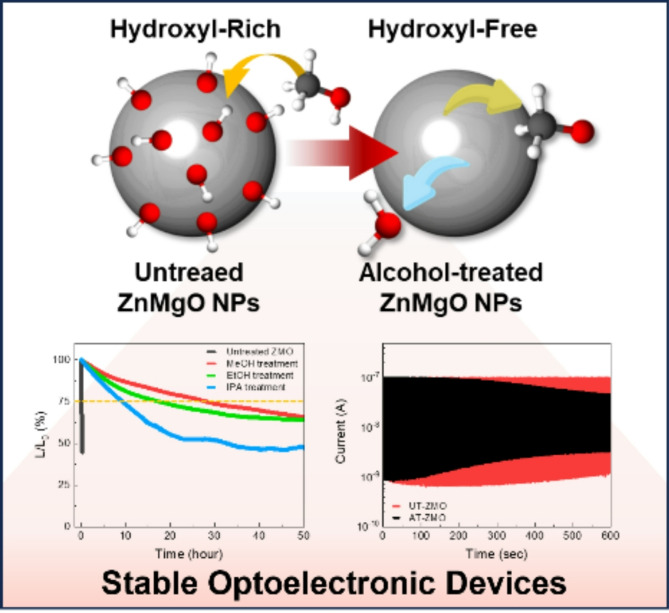

## Introduction

Quantum dots (QDs) have advanced significantly over the past several decades. Consequently, they have become promising materials for optoelectronic devices. Owing to their quantum confinement effect, the optical and electronic properties of QDs, such as their bandgap energy, can be precisely tuned by adjusting their size [1–6]. This tunability has led to extensive research in wide-bandgap applications, such as light-emitting diodes (LEDs) [7–15], and narrow-bandgap applications, such as solar cells and photodiodes (PDs) [16–30]. These properties have positioned QDs as key components for the development of next-generation display technologies, including QD LEDs (QLEDs) [31] and high-efficiency photovoltaics (PVs) [32]. The simultaneous development of efficient electron transport layers (ETLs) combined with QDs is essential for improving the performance of optoelectronic devices, such as LEDs, PVs, and PDs. Inorganic materials, such as ZnMgO nanoparticles (ZMO NPs), have garnered significant attention owing to their solution processability and potential to improve charge transport and device stability [33–38]. The solution processability of ZMO NPs simplifies fabrication and allows for cost-effective and scalable manufacturing processes, such as spin coating. Moreover, the compatibility of ZMO NPs with orthogonal solvents enables the formation of a multilayer device architecture without damaging the underlying layers. Thus, ZMO NPs are ideal for use in complex optoelectronic devices.

Efficient charge transport, including charge injection and extraction, is a primary factor in evaluating the performance of optoelectronic devices. To achieve high efficiency, the energy barrier for charge movement must be minimized. Moreover, charge loss during transport should be avoided. However, limitations such as low charge mobility in the charge transport layers, including ETLs and hole transport layers (HTLs), and the presence of trap sites at the surfaces and interfaces lead to significant charge loss and charge imbalance. These issues hinder efficient charge extraction and degrade overall device performance. Inorganic nanocrystals, which are widely used as ETLs, are considerably limited by their high surface energy, which arises from their nanoscale dimensions and large surface-to-volume ratio. High surface energy results in the formation of surface defects and trap sites, making them particularly susceptible to degradation upon exposure to humidity and oxygen, which ultimately compromises the stability and efficiency of optoelectronic devices [39–46]. The layers initially deposited in solution-processed optoelectronic devices are often damaged during the subsequent deposition. ZMO NPs readily adsorb hydroxyl groups (–OH) from ambient moisture owing to their strong polarity. This adsorption forms dipole moments and trap sites, disrupting charge transport and reducing both device efficiency and stability, which require effective passivation strategies to address [47–53].

In this study, an alcohol-based treatment method was developed to produce ‒OH-free ZMO NP thin films. Alcohol solvents, such as methanol (MeOH), ethanol (EtOH), and isopropanol (IPA), effectively remove surface ‒OH via hydrogen bonding, thereby reducing the number of charge trap sites and dipole moments and improving the stability of optoelectronic devices. Comprehensive analyses confirmed that the alcohol treatment (AT) successfully desorbed–OH from the surface of the ZMO NPs. This reduction in the number of surface charge trap sites directly enhanced the charge transport characteristics and electrical performance of the devices, enabling stable fabrication, even in external environments containing oxygen and moisture, without the need for inert gases such as nitrogen or argon. Moreover, the AT significantly improved the operational lifetime of the devices. The application of the proposed approach to QLEDs, whose stability is particularly affected by moisture, extended the device lifetime from 4 min under untreated (UT) conditions to 28 h under AT conditions, representing a substantial advancement in the stability and efficiency of QD-based optoelectronics. This study evaluates a generalizable and effective strategy for simultaneously enhancing the efficiency and stability of QLEDs and quantum dot photodiodes (QPDs) using ‒OH-free ZMO NPs enabled by alcohol-based surface passivation, offering a versatile solution applicable to various optoelectronic devices.

## Experimental

### Synthesis of PbS QDs

PbS QDs were synthesized using a modified version of a previously reported hot-injection method. In a 100 mL three-neck flask, 0.90 g lead oxide, 3 mL OA, and 20 mL 1-octadecene (ODE) were degassed under vacuum for 30 min. The solution was subsequently heated to a temperature range of 30–120 °C for 2 h until it became transparent. The solution was then treated at 120 °C under a nitrogen flow. A sulfur precursor solution, prepared by dissolving 240 µL bis(trimethylsilyl)sulfide in 8 mL ODE, was rapidly injected into the hot solution. After the injection, the mixture was allowed to cool to room temperature. The synthesized QDs were washed thrice with acetone and EtOH before being dispersed in octane at a concentration of 50 mg/mL.

### Synthesis of CdZnSeS/ZnS QDs

Green-emitting CdZnSeS core QDs were synthesized by combining 6.8 mmol zinc oxide, 0.28 mmol cadmium oxide, 14 mL OA, and 30 mL ODE in a 100 mL three-neck flask. The mixture was degassed at 110 °C for 30 min and subsequently heated to 320 °C under a nitrogen atmosphere. A selenium-sulfur (SeS) precursor solution, prepared by dissolving 3.2 mmol selenium and 4.8 mmol sulfur in 4 mL trioctylphosphine (TOP), was injected into the reaction mixture. The CdZnSeS cores were then grown at this temperature for 20 min. To grow the ZnS shell, 6.8 mmol zinc acetate dihydrate in 8 mL OA and 2 mL ODE was injected into the reaction, followed by a 10 min reaction at 275 °C. Subsequently, 20 mmol sulfur dissolved in 10 mL TOP was added at 275 °C and allowed to react for 10 min. The resulting CdZnSeS/ZnS core-shell QDs were purified using a hexane and EtOH mixture and then redispersed in hexane.

### Synthesis of ZnMgO NPs

ZnMgO NPs were synthesized using a colloidal method. In particular, 4.5 mmol zinc acetate dihydrate and 0.5 mmol magnesium acetate tetrahydrate were dissolved in DMSO (30 mL). Tetramethylammonium hydroxide (5 mmol) in 10 mL EtOH was then added dropwise to the solution under ice-cold conditions, and the reaction was continued for 1 h. The synthesized ZMO NPs were then precipitated and redispersed in EtOH using excess acetone.

### Fabrication of the PD

An ITO glass substrate was sequentially cleaned with acetone, isopropyl alcohol, and deionized water in an ultrasonic bath for 10 min each, followed by a 15 min UV (ultraviolet)-ozone treatment. ZnMgO NPs were deposited on the ITO substrates by spin-coating the ETL dispersion at 2500 rpm for 60 s, followed by two rinse-spin cycles with alcohol solvent at 3500 rpm for 30 s each, and annealing at 80 °C for 30 min. The PbS QDs were deposited layer-by-layer using spin coating. OA-capped PbS QDs were spin-coated at 2500 rpm for 30 s, followed by treatment with a TBAI solution (10 mg/mL in EtOH) for 30 s, two EtOH rinse-spin cycles, and annealing at 80 °C for 30 min. The TBAI coating process was repeated six times. Subsequently, the OA-capped PbS QDs were spin-coated again at 2500 rpm for 30 s, and an EDT solution (0.02 vol% in ACN) was applied to the PbS QD films for 30 s to induce ligand exchange. The films were washed twice with ACN and annealed at 80 °C for 30 min. This EDT treatment was repeated for two layers, with PbS-OA QDs spin-coated onto the PbS-EDT layers. Finally, a 60 nm layer of gold was deposited using thermal evaporation to form the top electrode.

### Fabrication of inverted LED

ITO substrates were cleaned according to the process used to fabricate the PDs. Subsequently, UV-ozone treatment was performed. ZnMgO NPs were spin-coated onto the ITO substrates at 3500 rpm for 60 s, followed by two rinse-spin cycles using an alcohol solvent at 3500 rpm for 30 s each and annealing at 80 °C for 30 min. To fabricate the emission layer, CdZnSeS@ZnS QDs were spin-coated onto the ITO/ETL substrates at 2000 rpm for 10 s. The organic materials and metals were sequentially deposited via thermal evaporation under a continuous vacuum. The HTL, comprising 4,4’-bis(carbazol-9-yl)biphenyl (CBP), was thermally evaporated at a deposition rate of 1 Å/s. MoO_3_ and the aluminum electrode were also thermally evaporated at 0.5 and 2 Å/s, respectively.

### Characterization

The current–voltage characteristics were measured using a source meter (Keithley 2400, Tektronix). The current density–voltage–luminance characteristics of the devices were measured under ambient conditions using a spectroradiometer (Minolta CS2000) equipped with a Keithley 2400 source meter. The PDs were measured using a 980 nm laser (MDL-IIIR-980/500mW, Changchun New Inc.). The structural properties of the PDs were investigated using FT-IR spectroscopy (Nicolet iS50, Thermo Scientific). UPS and XPS (Nexsa, Thermo Fisher Scientific) with He I radiation (21.22 eV) were employed to determine the E_f_ and valence bands, respectively. The secondary cut-off in the UPS spectra was used to analyze the work functions (WFs) of the materials using the equation WF = 21.22 eV ‒ secondary cut-off. The difference between the valence band maximum and E_f_ was determined by curve fitting in the low-binding energy region. The surface morphologies were examined using AFM (XE7, Park Systems). The optical properties of the ZnMgO NPs were analyzed by UV-visible (V-770, JASCO) and PL spectroscopy (FP-8500, JASCO). The water contact angles of the surfaces were determined using a contact angle goniometer (Phoenix 150; SEO).

## Results and discussion

**Fig. 1:**
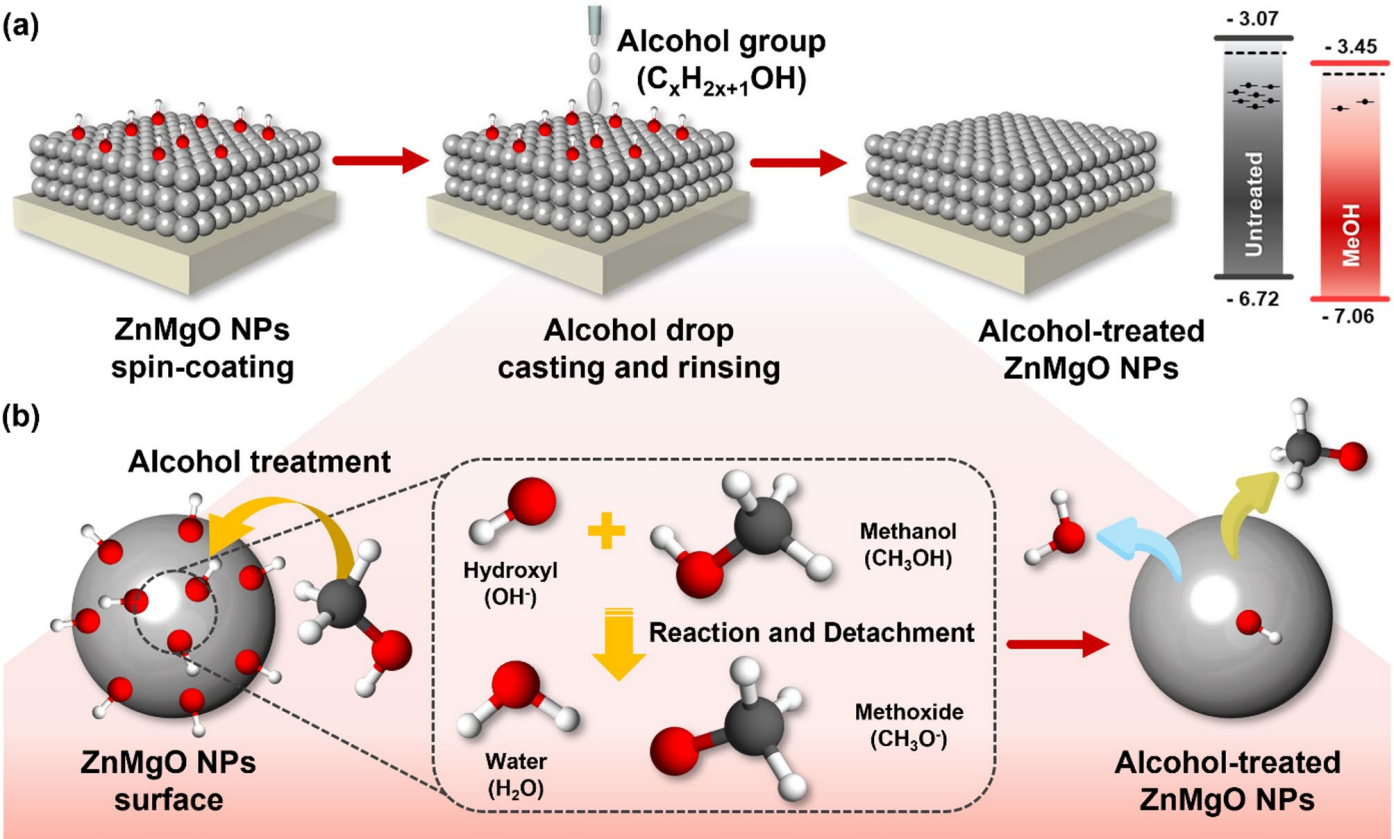
Schematics illustrating (**a**) removing process of ‒OH from ZMO NPs using AT and (**b**) surface reactions on the ZMO NPs before and after AT

Figure 1 schematically illustrates the treatment process and chemical reactions involved in the removal of –OH from the surface of the ZMO NPs using AT. Figure 1**(a)** shows the stepwise process of ‒OH removal from the spin-coated ZMO NPs using AT. In the first step, ZMO NPs were uniformly spin-coated onto an indium tin oxide (ITO) substrate, forming a thin NP layer with abundant ZMO-OH on the surface. In the second step, ZMO NPs were drop-cast and rinsed with alcohol solvents such as MeOH, EtOH, and IPA. This step facilitates the interaction between ‒OH and the alcohol molecules. The AT successfully detached the surface ‒OH, leaving behind a clean ZMO surface with a significantly reduced ‒OH content. The AT restores the surface to an optimal state for charge transport in optoelectronic devices. The surface-bound ‒OH possesses strong polarity, which induces charge traps that impede the movement of electrons and holes, leading to undesirable leakage currents [40, 41].

Figure 1**(b)** schematically illustrates the chemical mechanism underlying the removal of ‒OH from the surface of the ZMO NPs during the AT. ‒OH are initially bound to the surface through interactions with positively charged metal ions (ZnMgO^+^),

1 $$\:{\text{Z}\text{n}\text{M}\text{g}\text{O}}^{+}\:+\:{\text{O}\text{H}}^{-}\:\to\:\:\text{Z}\text{n}\text{M}\text{g}\text{O}-\text{O}\text{H}$$

Once attached, –OH readily interacts with the conduction band (CB) electrons because of their strong electronegativity, forming a charge-trapped state,

2 $$\:\text{Z}\text{n}\text{M}\text{g}\text{O}-\text{O}\text{H}\:+\:{\text{e}}^{-}\:\to\:\:\text{Z}\text{n}\text{M}\text{g}\text{O}-{\text{O}\text{H}}^{-}$$

The charge-trapped state degrades the electronic properties of the ZMO layer and increases its surface dipole moment, leading to a diminished device performance.

Treatment with MeOH causes –OH to undergo a proton transfer reaction, forming water and detaching from the ZMO surface,

3 $$\:\text{Z}\text{n}\text{M}\text{g}\text{O}-\text{O}\text{H}\:+\:{\text{C}\text{H}}_{3}\text{O}\text{H}\:\to\:\:\text{Z}\text{n}\text{M}\text{g}\text{O}\:+\:{\text{H}}_{2}\text{O}\:+\:{\text{C}\text{H}}_{3}{\text{O}}^{-}$$

The CH_3_O^−^ ion produced as a byproduct during this reaction helps balance the overall reaction system charge. As a strong base, CH_3_O^−^ remains in the surrounding solvent where it can participate in further reactions such as neutralizing residual hydroxide ions (OH^-^) or regenerating CH_3_OH through interactions with water,

4 $$\:{\text{C}\text{H}}_{3}\text{O}-\:+\:{\text{H}}_{2}\text{O}\:\to\:\:{\text{C}\text{H}}_{3}\text{O}\text{H}\:+\:{\text{O}\text{H}}^{-}$$

The presence of CH_3_O^−^ thus contributes to maintaining the chemical equilibrium in the system while also ensuring that ‒OH are effectively removed without accumulating unwanted byproducts. This process eliminates surface ‒OH and reduces surface dipoles, thus improving the electronic properties of the ZMO layer by eliminating trap sites and restoring its clean, functional surface.

**Fig. 2:**
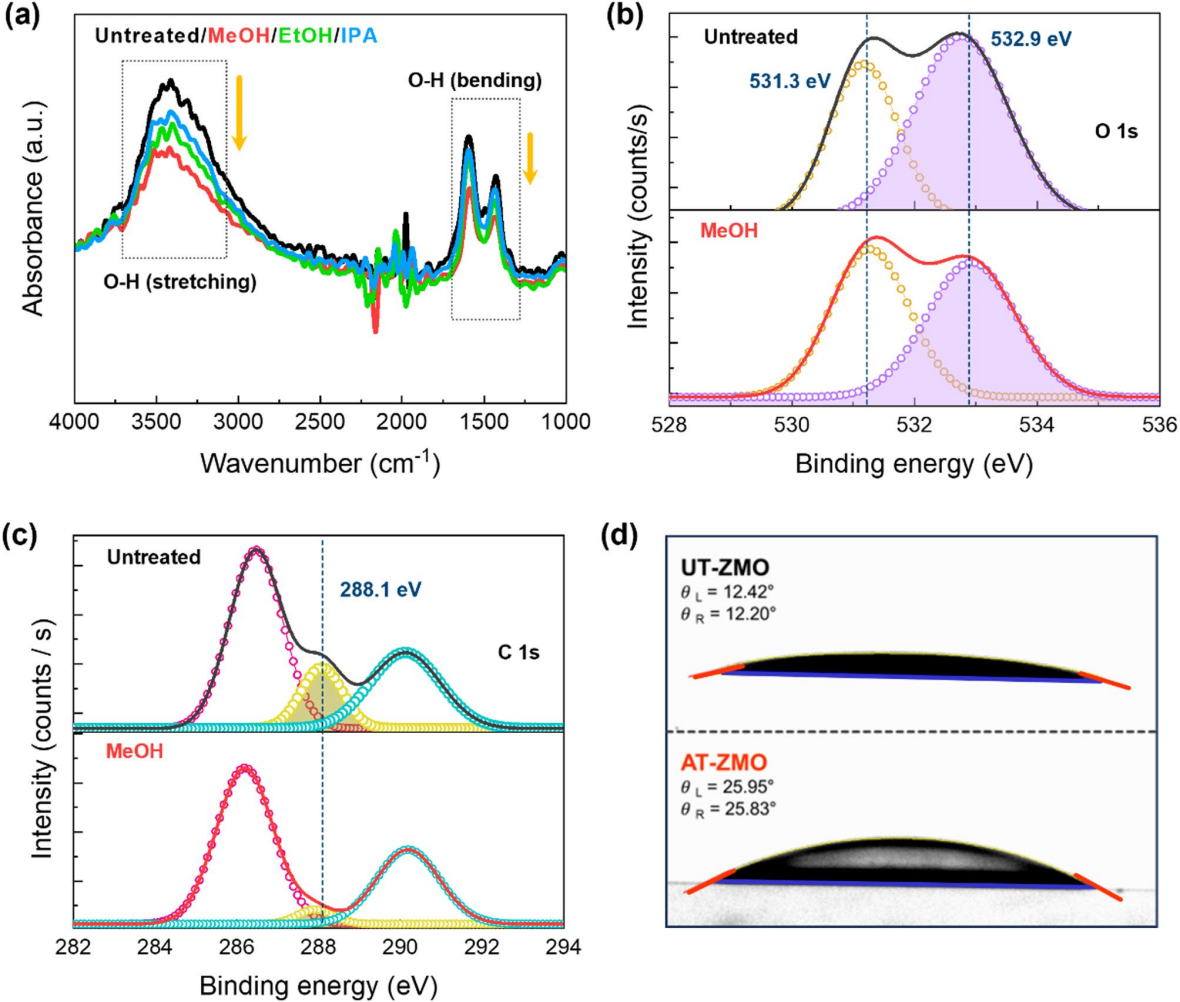
(**a**) FT-IR spectra of UT-ZMO and MeOH-, EtOH-, and IPA-treated ZMO. XPS high-resolution (**b**) O 1s and (**c**) C 1s photoelectron peaks of UT-ZMO and MeOH-treated ZMO. (**d**) Contact angle images of the UT- and AT-ZMO NPs (UT-ZMO and AT-ZMO, respectively)

Figure 2 provides a comprehensive overview of the optical and chemical properties of UT-ZMO and AT-ZMO. The figure shows the removal of the surface ‒OH groups and the resulting changes in the material characteristics. The FT-IR spectra of UT-ZMO and AT-ZMO in Fig. 2**(a)** show a decrease in ‒OH absorbance on the surface of the ZMO NPs coated on the glass substrates. The peaks at 3200‒3700 and 1250–1750 cm^-1^, which exhibit the highest intensity under UT conditions, correspond to O-H stretching and bending vibrations, respectively. However, AT decreased the intensities of these peaks, with MeOH inducing the most pronounced reduction, followed by EtOH and IPA. This trend was attributed to the higher polarity and smaller molecular size of MeOH, which facilitated more rapid and effective interactions with the NP surface. Although all alcohols form hydrogen bonds, smaller molecules, such as those in MeOH, are more reactive because of their greater ability to access and interact with surface sites. Fig. S1 compares the FT-IR spectra of highly polar solvents, such as methyl acetate (MeOAc), acetonitrile (ACN), and water, under UT conditions. In particular, under UT conditions, ‒OH remained on the surface of the ZMO NPs treated with both MeOAc and ACN, as these solvents, despite their high polarity, were unable to donate protons or establish strong hydrogen bonds with ‒OH. In contrast, treatment with water results in almost complete removal of ‒OH owing to its strong hydrogen-bonding capability and ability to facilitate proton transfer reactions. This indicates that solvents capable of forming hydrogen bonds, such as water, interact more effectively with ‒OH than purely polar solvents, thereby enhancing their removal.

Figures 2**(b) and 2(c)** show X-ray photoelectron spectroscopy (XPS) profiles of UT-ZMO and AT-ZMO. The O 1s peaks depend significantly on the solvent used. Figure 2**(b)** shows that the O 1s peaks for both UT-ZMO and AT-ZMO could be deconvoluted into two components centered at 531.3 and 532.9 eV, corresponding to lattice oxygen and hydroxide oxygen (O_OH_), respectively. The O_OH_-associated peak area ratio decreased from 54.1% in UT-ZMO to 47.6% in AT-ZMO, indicating that MeOH-based surface functionalization effectively minimized the ‒OH defects on the ZMO NPs, which was likely due to a reaction between the surface ‒OH and alcohol groups that released H_2_O (Eq. (3)). Figure 2**(c)** shows the C 1s peaks for UT-ZMO and AT-ZMO, which were deconvoluted into three components at 286.3, 288.1, and 290.4 eV, corresponding to C-C, C-O, and O-C = O, respectively. The C-C and O-C = O ratios for UT-ZMO and AT-ZMO were 56.60% and 63.75%, respectively, and 24.12% and 30.33%, respectively. The C-C to O-C = O ratio remained consistent at approximately 7:3 under both conditions. In contrast, the C-O component decreased significantly from 19.28% in UT-ZMO to 5.91% in AT-ZMO, suggesting that the AT removed ‒OH and the acetate residues originating from the zinc acetate dihydrate used during the ZMO NP synthesis. Similar trends were observed in the O 1s and C 1s XPS spectra of the EtOH- and IPA-treated ZMO, as shown in Fig. S2.

Figure 2**(d)** shows that the contact angle for UT-ZMO increased from 12.31° to 25.89° after treatment with MeOH. This increase implies a reduced surface wettability, indicating a significant change in the surface energy. The wetting work (*W*), which represents the interaction between the liquid and solid surfaces, is defined by the Young-Dupre equation, as follows:

5 $$\:W={\gamma\:}_{LV}(1+cos\theta\:),$$

where $\:{\gamma\:}_{LV}$ is the liquid surface tension, and $\:\theta\:$ is the contact angle. Equation (6) was used to estimate the relative change in the surface energy ($\:{\gamma\:}_{SV}$) induced by AT as follows:

6 $$\:\frac{{\gamma\:}_{SV},\:after}{{\gamma\:}_{SV},\:\:before}=\:\frac{1+cos{\theta\:}_{after}}{1+cos{\theta\:}_{before}\:}.$$

Substituting the measured angles (cos 12.31° ≈ 0.9784 and cos 25.89° ≈ 0.9004), the surface energy after the AT was calculated to be approximately 96% of the original value, representing a 4% decrease. This reduction in the surface energy, particularly in its polar component ($\:{\gamma\:}_{SV}^{p}$), is attributed to the removal of hydrophilic ‒OH during the AT. These changes, as confirmed by spectroscopic evidence, demonstrate that the AT effectively modified the ZMO surface, reducing its hydrophilicity and increasing its hydrophobicity, which is advantageous for optoelectronic applications.

**Fig. 3:**
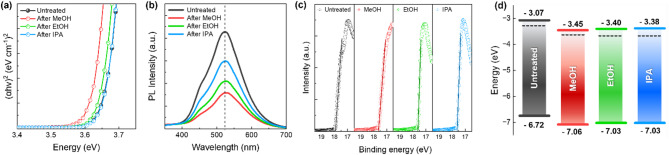
(**a**) Absorption spectra of UT-ZMO and AT-ZMO from the Tauc plot analysis. (**b**) PL spectra of UT-ZMO and MeOH-, EtOH-, and IPA-treated ZMO. (**c**) UPS spectra showing the high-binding-energy secondary electron cut-off regions. (**d**) Band diagram of UT-ZMO and AT-ZMO

Figure 3**(a)** shows the bandgap variations in the ZMO NPs after the AT from the Tauc plot. The bandgap values under UT conditions are 3.65 eV, decreasing to 3.61, 3.63, and 3.65 eV after treatment with MeOH, EtOH, and IPA, respectively. These results indicate that changing the solvent from IPA to MeOH slightly narrows the bandgap. The observed trend is attributed to the higher reactivity of MeOH, which originates from its smaller molecular size. This AT reduces the density of surface-related defect states and dipole moments, thereby suppressing the sub-bandgap absorption, leading to a more defined optical band edge, as reflected in the slightly increased bandgap values extracted from the Tauc plots. The reduction in the surface oxygen vacancies (V_O_) in the ZMO NPs following alcohol functionalization is evidenced by a significant decrease in the V_O_-associated PL intensity, as shown in Fig. 3**(b)**. The PL intensity of the MeOH-treated ZMO, as compared with that of UT-ZMO, exhibited a 62.8% reduction accompanied by a 2.7 nm blue shift in the main emission peak. The PL spectrum of the ZMO NPs primarily originated from the trap states associated with the V_O_ within the NPs. The AT further passivates the surface of the ZMO NPs by filling the surface V_O_ sites, effectively reducing the V_O_-induced trap states and eliminating the hydroxyl-related defects [40, 41]. This comprehensive defect passivation results in the observed optical properties.

Furthermore, Fig. 3**(c)** shows the shift in the Fermi level (E_f_) of the ZMO NPs following the AT, as analyzed using ultraviolet photoelectron spectroscopy (UPS) in the secondary cut-off region. The secondary cut-off for UT-ZMO occurs at 17.91 eV, while those for AT-ZMO (MeOH, EtOH, and IPA) shift to 17.63, 17.62, and 17.62 eV, respectively, representing a difference of approximately 0.3 eV. The CB minimum and valence band maximum, calculated from the valence band edge region and Tauc plot, are shown in Fig. S3 and summarized in Table S1. The shift in E_f_ is attributed to changes in the dipole moment caused by the presence and subsequent removal of ‒OH from the surface of the ZMO NPs. The surface ‒OH prior to the AT generates dipole moments that position the E_f_ at − 3.29 eV, likely due to the formation of electron trap sites. The removal of ‒OH by the MeOH treatment reduces the dipole moments and eliminates trap sites, downshifting the E_f_ to − 3.57 eV.

Figure 3**(d)** summarizes the bandgap changes in the ZMO NPs before and after AT. The UT-ZMO CB maximum (CBM) was positioned at − 3.07 eV while the E_f_ was positioned at − 3.29 eV, creating a 0.22 eV gap. The CB and E_f_ of MeOH-treated ZMO shifted to − 3.45 and − 3.57 eV, respectively, indicating a downshift of the band with decreasing surface dipole moments and the number of trap sites, thereby enhancing the n-type characteristics. Although the EtOH and IPA treatments similarly downshifted the bandgap and reinforced the n-type behavior, the shift was less pronounced than the treatment with MeOH, consistent with the FT-IR, XPS, and contact angle results, which confirms the superior efficacy of MeOH in removing ‒OH from the ZMO surface. As summarized in Table S2, the AT process provides a simpler and more scalable alternative to conventional ZMO passivation methods, which often require more complex chemistry or equipment.

**Fig. 4:**
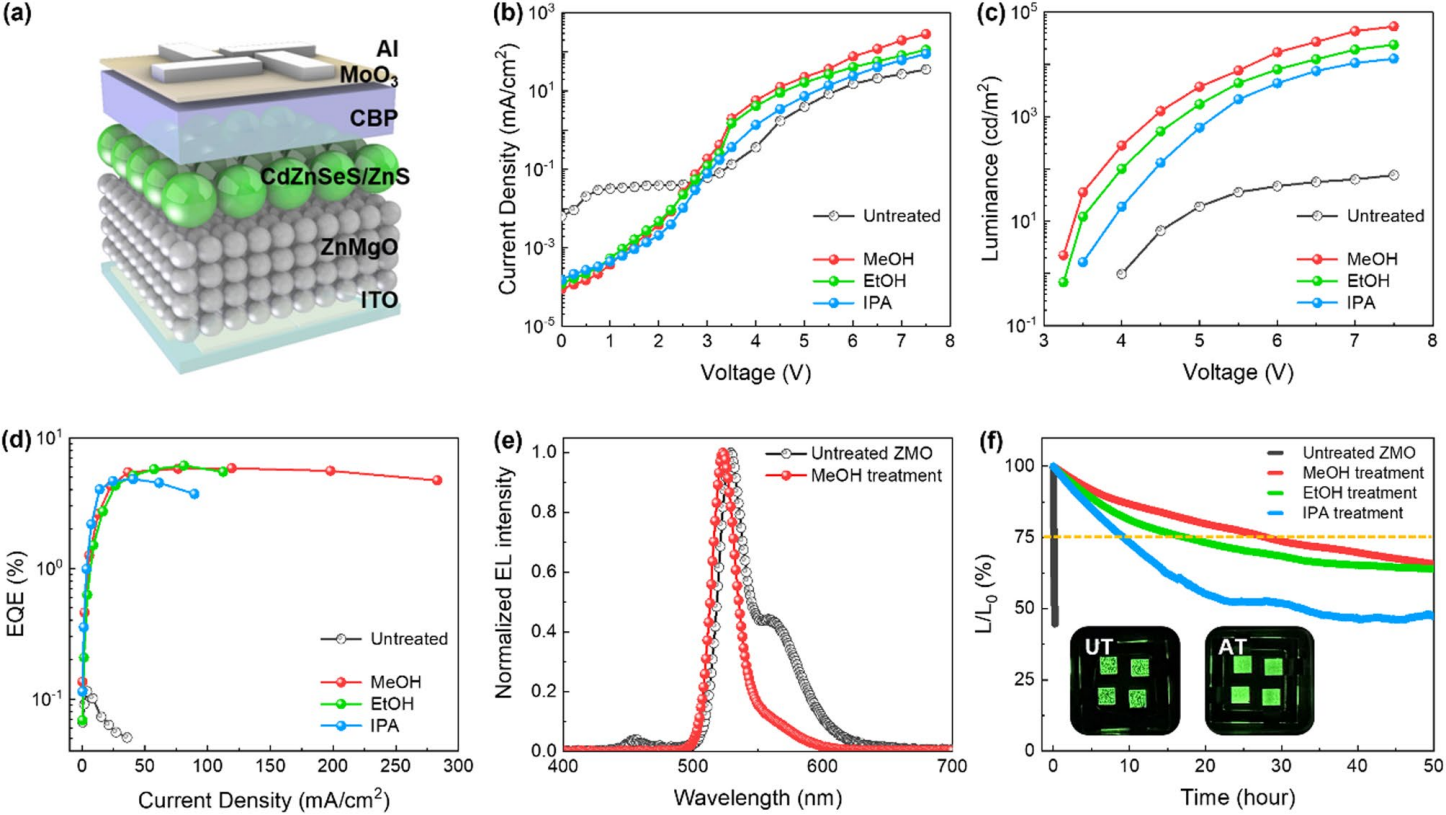
(**a**) Schematic illustrating the device structure of QLEDs incorporating UT-ZMO and AT-ZMO. (**b**) Current density‒voltage characteristics, (**c**) luminance‒voltage, and (**d**) EQE-voltage curves of UT-ZMO and the MeOH-, EtOH-, and IPA-treated ZMO. (**e**) Comparison of the EL spectra of UT-ZMO and MeOH-treated ZMO QLEDs. (**f**) Comparison of the operational stabilities of UT-QLED and AT-QLED

Figure 4 compares the performance of the AT-QLEDs. The QLEDs were fabricated in an inverted structure under ambient air conditions using an ITO/AT-ZMO NPs/CdZnSeS@ZnS/CBP/MoO_3_/Al configuration to isolate the effects of the ZMO NP surface modification, as shown in Fig. 4**(a)**. Figure 4**(b)** shows the current density–voltage characteristics of the QLEDs incorporating AT-ZMO. In the 0–3 V range, the current density in the UT-QLED is 6.42 × 10^− 3^ mA/cm^2^ at 0 V, whereas those in the MeOH-, EtOH-, and IPA-treated QLEDs are significantly lower (8.90 × 10^− 5^, 1.21 × 10^− 4^, and 1.52 × 10^− 4^ mA/cm^2^, respectively). The 72-fold difference between the current densities of the UT-ZMO and MeOH-treated QLEDs is attributed to the ‒OH on the surface of the ZMO NPs, which acts as trap sites that decrease the electron transport properties [41]. The AT removes OH⁻, which eliminates the trap sites and maintains a lower current density. The current density of the AT-QLEDs at voltages exceeding 3 V was lower than that of UT-QLED because of the inhibited electron injection and transport caused by the remaining trap sites, which limited electron transport. Moreover, electron-only devices (EODs) were fabricated for comparison to investigate the properties of the ZMO NPs. Fig. S4 shows that MeOH-treated ZMO exhibited the lowest current among all EODs. Among the AT-QLEDs, the MeOH-treated device exhibited the highest current density, followed by the EtOH- and IPA-treated devices. This trend is consistent with the FT-IR results, where MeOH exhibited the greatest reduction in OH⁻, thereby removing excess trap sites on the surface of the ZMO NPs and enhancing the electron transport.

Figure 4**(c)** compares the luminance characteristics of the UT-QLED with those of the AT-QLEDs. The turn-on voltage in the UT-QLED was 4 V with a luminance of 1 cd/m^2^, whereas the MeOH-, EtOH-, and IPA-treated QLEDs exhibited significantly higher luminance values of 282, 131, and 19 cd/m^2^, respectively, at 4 V with a further reduction in the turn-on voltage. The UT-QLED exhibited a maximum luminance of only 76 cd/m^2^ at 7.5 V, whereas the MeOH-, EtOH-, and IPA-treated QLEDs exhibited maximum luminance values of 53 595, 23 901, and 12 924 cd/m^2^, respectively. The superior luminance of the MeOH-treated QLED is attributed to the reduced number of trapped electrons and the higher proportion of radiative recombination of electrons transported to the QDs, as opposed to those remaining in the trap sites. The current density and luminance of the AT-QLEDs exhibit a similar correlation, as shown in Fig. 4**(d)**, which also reveals a consistent EQE trend. The UT-QLED exhibited a lower luminance relative to its current density, resulting in a significant decrease in EQE with increasing current density, exhibiting a value as low as 0.1%.

Figure 4**(e)** shows the normalized EL spectra at the turn-on voltage. The EL spectrum of the UT-QLED exhibited two peaks: a primary CdZnSeS@ZnS emission peak at 529 nm and an additional emission peak at 558 nm. The AT-QLEDs exhibited a single emission peak centered at 523 nm. As shown in Fig. 2**(d)**, the broad PL spectrum of the ZMO NPs, ranging from 520 to 550 nm, is attributed to V_O_. Therefore, the secondary emission peak in the UT-QLED EL spectrum originated from the ZMO NPs. This indicates that the electrons trapped on the ZMO surface cannot reach the QDs for radiative recombination. Instead, the holes transported from the HTL recombined with the trapped electrons at the ZMO NP interfaces, resulting in additional emission. Fig. S5 shows that the AT of the ZMO NPs leads to reduced exciton quenching in the QDs.

Figure 4**(f)** shows the operational lifetimes of the UT-QLED and AT-QLEDs at a luminance of 500 cd/m^2^. The UT-QLED lifetime decreased rapidly, reaching T_75_ within 4 min. In contrast, the MeOH-, EtOH-, and IPA-treated QLEDs exhibited significantly extended lifetimes of 28 h, 17 h, and 9 h, respectively, indicating enhanced stability. These results suggest that the OH⁻ on the surface of UT-ZMO reduces the device efficiency and directly impacts the device stability and longevity. The AT effectively maintained device stability over extended operational periods. Table S3 presents a comparison of the previously reported ZMO passivation approaches and their corresponding QLED lifetimes. The results demonstrate the competitive performance of AT-QLEDs under ambient conditions. The inset images in Fig. 4**(f)** show that UT-QLED exhibited uneven EL across its surface with visible residual patterns, whereas the AT-QLEDs exhibited uniform EL with a clean residue-free emission profile, demonstrating superior surface uniformity.

**Fig. 5:**
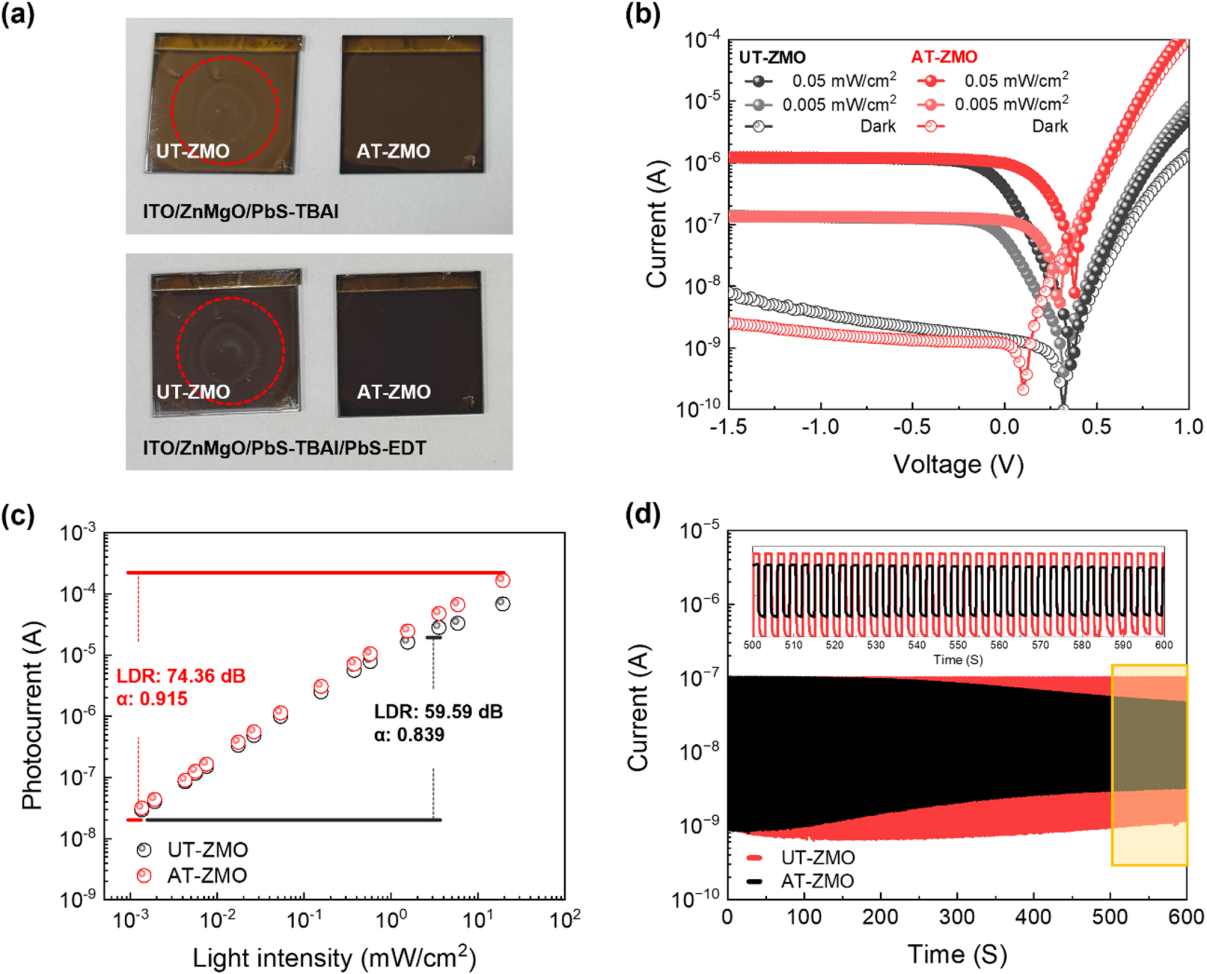
(**a**) Images showing PbS QDs coated on the UT-ZMO (left) and AT-ZMO (right) NP surfaces. (**b**) Current‒voltage characteristics of the UT-ZMO and AT-ZMO QPDs under dark conditions and two different NIR illumination intensities (0.005 and 0.05 mW/cm²). (**c**) Photocurrent as a function of light intensity, and (**d**) current as a function of time for the UT-ZMO and AT-ZMO QPDs

PDs with AT/PbS-tetrabutylammonium iodide (TBAI)/PbS-1,2-ethanedithiol (EDT)/Au-structured ITO/ZnMgO NPs were fabricated. Figure 5 shows a comparison of the performance of the UT- and MeOH-treated PbS-QD-based PDs with a narrow bandgap suitable for infrared light absorption. Figure 5**(a)** shows the morphology of the thin films after coating the ZMO NPs with the PbS QDs. The deposition of the PbS QDs, which were capped with oleic acid (OA), onto the UT-ZMO NPs initiated a ligand exchange reaction with TBAI, forming a film with visible residue patterns of the originally deposited PbS QDs. However, the application of the same process to the AT-ZMO NPs resulted in a clean, uniform coating without visible marks or residues. After coating the HTL with PbS-EDT, circular marks from the initial PbS QD deposition remained on the UT-ZMO NPs, affecting the thin-film morphology. However, the treated ZMO NPs maintained their intact morphology with no signs of disruption. These observations suggest that the residue patterns and delamination effects observed on the UT-ZMO NPs originate from the interactions between the PbS QDs and the ZMO NP surface during layer-by-layer deposition. Atomic force microscopy (AFM) measurements further demonstrated that the roughness of UT-ZMO was 56.0 nm, which is attributed to the residue and reactants, whereas MeOH-treated ZMO exhibited a roughness of 4.8 nm, as shown in Fig. S6, indicating the formation of a high-quality thin film.

Figure 5**(b)** shows the current–voltage characteristics of the UT- and MeOH-treated QPDs. UT QPD exhibits a dark current of 7.92 × 10^− 9^ A at − 1.5 V, whereas the MeOH-treated QPD exhibits a reduced dark current of 2.54 × 10^− 9^ A at the same voltage, indicating suppression of the dark current. The zero-crossing voltage (I = 0) of the UT QPD (+ 0.32 V) shifted closer to equilibrium in the MeOH-treated QPD (+ 0.10 V), yielding a 0.22 V reduction in the dark state. This shift strongly supports the removal of dipole-forming surface species and mobile ions through AT, as further evidenced by the comprehensive surface chemical and electronic structure analyses presented in Figs. 2 and 3. The detailed forward and reverse sweep I–V characteristics, along with the extracted zero-crossing voltages, are provided in Fig. S7, which clearly shows the reduced hysteresis after AT. The photocurrents of both QPDs were measured under IR illumination at two 980 nm intensities, namely, 0.005 and 0.05 mW/cm^2^. At 0 V, UT QPD exhibited EQE values of 31.95% and 22.63%, respectively, with increasing IR intensity, whereas the MeOH-treated QPD exhibited significantly higher values of 64.77% and 58.31%, respectively. The EQE values for the UT- and MeOH-treated QPDs at − 1 V were 72.32% and 70.98%, respectively, and 74.30% and 71.92%, respectively. These results suggest that charge extraction was hindered in UT QPD because of the presence of ‒OH on the surface of the ZMO NPs, particularly at 0 V, where the lower EQE values highlight the effect of trapped charges. The high dark current, shifted zero-bias voltage, and reduced EQE under IR illumination in UT QPD can be attributed to charges trapped on the surface of the ZMO NPs due to ‒OH, which restricts electron transport and extraction, thereby reducing the device efficiency and sensitivity.

Figure 5**(c)** shows that QPDs exhibit a linear relationship between the photocurrent and incident light power over a range from approximately 1.0 µW/cm^2^ to 10 mW/cm^2^. The linear dynamic range (LDR) of the photodetectors calculated using this relationship significantly increased from 59.59 dB (UT QPD) to 74.36 dB (MeOH-treated QPD). These results suggest that a broader detection range was obtained in the self-powered mode. The slopes of the photocurrent as a function of the light intensity for the UT and MeOH-treated QPDs were 0.839 and 0.915, respectively, suggesting that the AT-ZMO ETL effectively mitigated the current losses due to charge trapping. This improvement in the photo-extraction efficiency and expanded LDR emphasizes the role of AT-ZMO in stabilizing charge transport and optimizing the photodetector performance.

Figure 5**(d)** shows the time-resolved current responses of the UT- and MeOH-treated QPDs under repeated dark and IR illumination cycles. The dark current in the UT QPD sharply increased after 100 s in the off state, whereas the photocurrent under IR illumination gradually decreased after approximately 200 s. In contrast, the MeOH-treated QPD maintained a stable dark current of 1‒2 × 10^− 9^ A during the off state and consistently maintained a photocurrent of approximately 1 × 10^− 7^ A under IR illumination without noticeable degradation. The fluctuations in the dark current and photocurrent of UT-QPD suggest accelerated device degradation owing to the presence of ‒OH on the surface of the ZMO NPs, which impedes electron transport and drastically reduces the operational lifetime of the device, preventing it from achieving its full performance potential. Consistent with the AT-QLEDs, the MeOH-treated QPD suppressed degradation through bandgap stabilization and the removal of trap sites, which enhanced the overall robustness and stability of the device (Fig. S8).

## Conclusion

A detailed analysis of the mechanism governing the removal of surface-bound ‒OH from ZMO NPs by the AT demonstrated that ‒OH was removed through hydrogen bonding and the subsequent formation of water and alkoxide byproducts. MeOH, which had the smallest molecular size among the alcohols tested, was the most efficient in facilitating ‒OH desorption. This ‒OH removal process reduces the number of charge trap sites, improves electron transport, and enables stable device operation, even in oxygen- and moisture-rich environments. The AT enhanced the electron transport properties, efficiency, and operational stability of the QLEDs and QPDs, thereby demonstrating its potential as a surface engineering method for overcoming critical limitations in optoelectronic devices.

## Electronic supplementary material

Below is the link to the electronic supplementary material.

Supplementary Material 1

## Data Availability

The datasets used in this study are available from the corresponding author on reasonable request.
