# Supplementary material for: Surface passivation engineering for stable optoelectronic devices via hydroxyl-free ZnMgO nanoparticles
Source: Nano Converg. 2025 Jun 9;12:28. doi: 10.1186/s40580-025-00493-2 (PMC12149040; doi:10.1186/s40580-025-00493-2)
Supplement: Supplementary file 1 — Supplementary Material 1 [file 40580_2025_493_MOESM1_ESM.docx]

**Supplementary materials**

**Surface Passivation Engineering for Stable Optoelectronic Devices via Hydroxyl-Free ZnMgO Nanoparticles**

Seongkeun Oh^1‡^, Jaehwi Choi^2‡^, Junhyeok Park^1^, Young Kyun Choi^1^, Taesung Park^1^, Awais Ali^1^, Junhyuk Ahn^1^, Jiwan Kim^2^*, and Soong Ju Oh^1^*

^1^Department of Materials Science and Engineering, Korea University, 02841, Republic of Korea

^2^Department of Advanced Materials Engineering, Kyonggi University, 16227, Republic of Korea

**Table S1.** Energetic information on a series of HC-ZMO and AC-treated ZMO NP films obtained from the UPS and absorption spectra.

| **Film sample** | **Secondary**  **cut-off (eV)** | **E_f_ (eV)** | **Valence band**  **onset (eV)** | **Valence band maximum**  **(eV)** | **Bandgap**  **(eV)** | **CBM**  **(eV)** |
| --- | --- | --- | --- | --- | --- | --- |
| UT  ZMO | 17.91 | 3.29 | 3.43 | 6.72 | 3.65 | 3.07 |
| MeOH-treated  ZMO | 17.63 | 3.57 | 3.49 | 7.06 | 3.61 | 3.45 |
| EtOH-treated  ZMO | 17.62 | 3.58 | 3.45 | 7.03 | 3.63 | 3.40 |
| IPA-treated  ZMO | 17.62 | 3.58 | 3.45 | 7.03 | 3.65 | 3.38 |

**Table S2.** Comparison of the ZnMgO passivation methods

| **Methode** | **Key Principle** | **Process**  **Complexity** | **Equipment/Material**  **Requirement** | **Effectiveness**  **(Trap, Dipole Removal)** |
| --- | --- | --- | --- | --- |
| **Alcohol Treatment**  **(This work)** | Remove hydroxyls  *via* solvent rinsing | Low | Common solvents  (MeOH, EtOH, IPA) | High |
| UV-Ozone  Treatment | Oxidizes/removes organics  and surface states | Medium | UV-ozone lamp,  Controlled exposure | High |
| Chemical  Functionalization | Adds passivating ligands | Medium-High | Functional molecules,  synthesis steps | High |
| Ligand  Exchange | Replaces surface ligands  With stable coordinating groups | High | Tailored ligand,  Exchange protocols | Very High |

**Table. S3.** Comparison of QLED lifetime according to representative ZnMgO passivation strategies

| **Passivation**  **Strategy** | **Process-**  **environment** | **Initial Luminance**  **(cd/m^2^)** | **Lifetime**  **Metric** | **Value**  **(hour)** | **Reference** |
| --- | --- | --- | --- | --- | --- |
| Alcohol Treatment-ZMO | Air | 500 | T_75_ | 28 | This work |
| ZnO/UVO/ZMO- UVO treatment | Glovebox-N_2_ | 100 | T_50_ | 30 | [1] |
| Acrylate-functionalized ZMO | Glovebox-N_2_ | 250 | T_50_ | 35.8 | [2] |
| HEMA-premixed ZMO | Glovebox-N_2_ | 2000 | T_50_ | 3.6 | [3] |
| Hexane/IPA treatment ZMO | Glovebox-N_2_ | 150 | T_70_ | 8.4 | [4] |

**
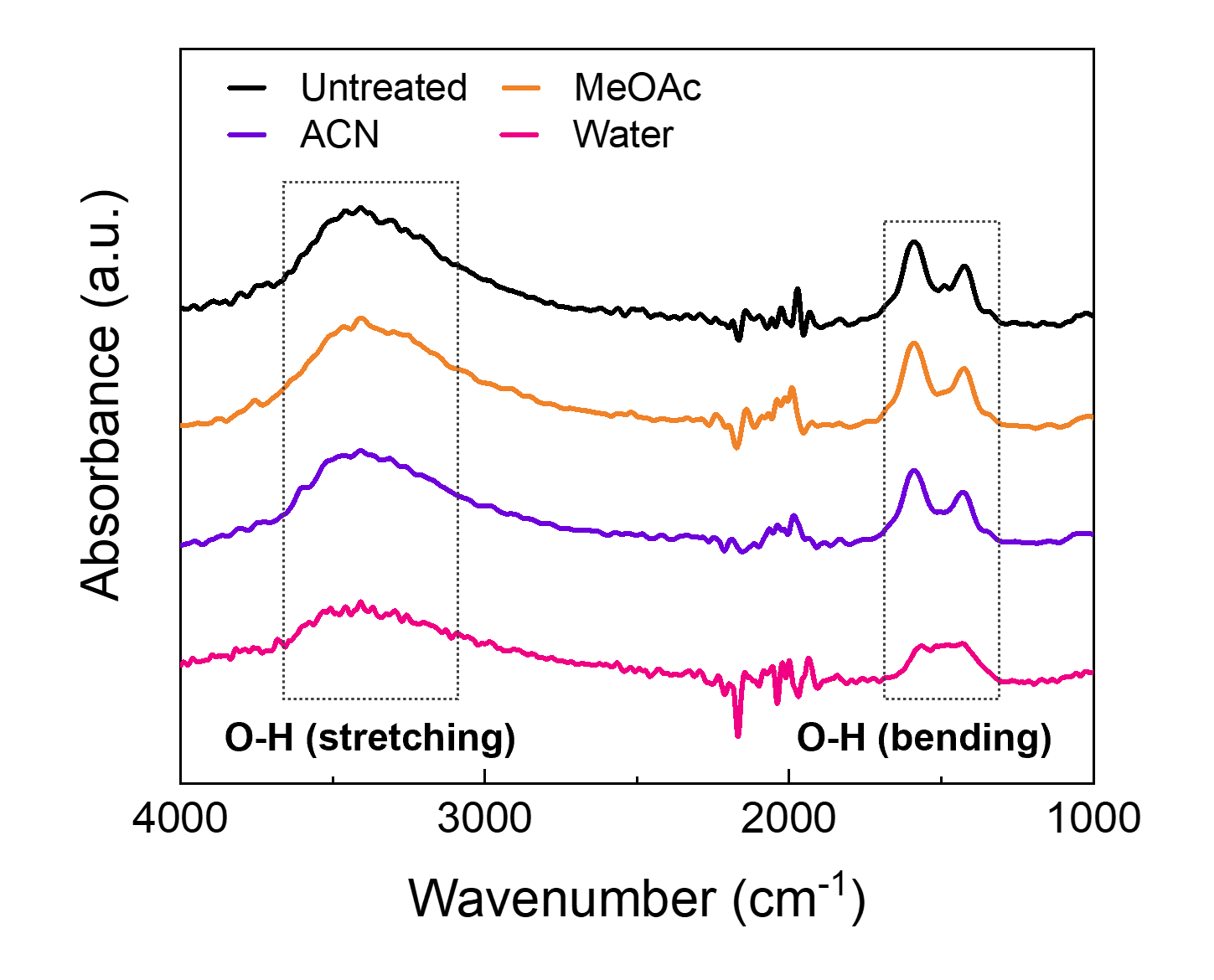
**

**Fig. S1.** FT-IR spectra of the ZMO NPs under humid conditions and after treatment with high polarity solvents (MeOAc, ACN, and water).


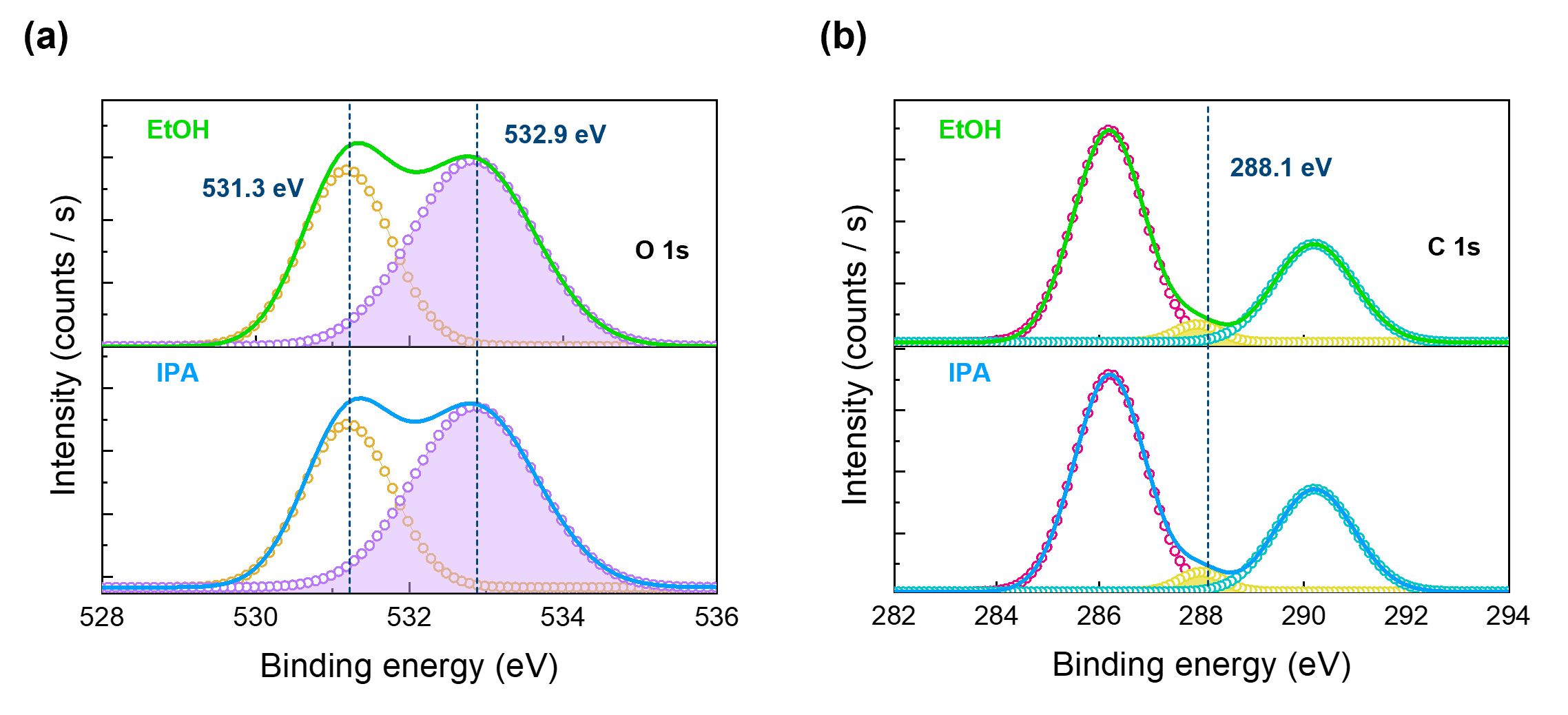


**Fig. S2.** XPS high-resolution (a) O 1s and (b) C 1s photoelectron peaks of EtOH- and IPA-treated ZMO.


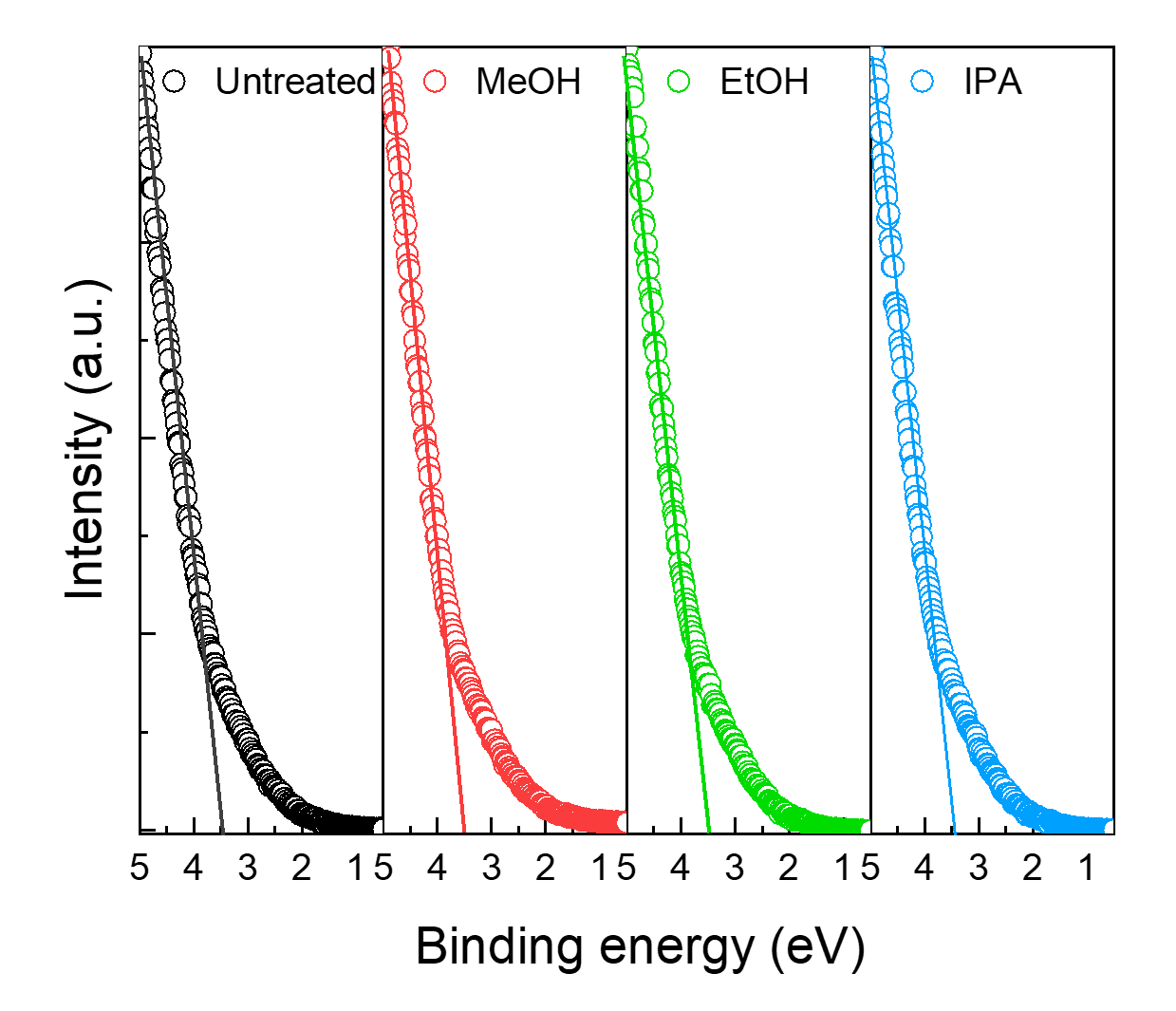


**Fig. S3.** UPS spectra showing the high-binding energy valence band onset regions of UT-ZMO (black) and MeOH- (red), EtOH- (green), and IPA-treated ZMO (blue) nanofilms.


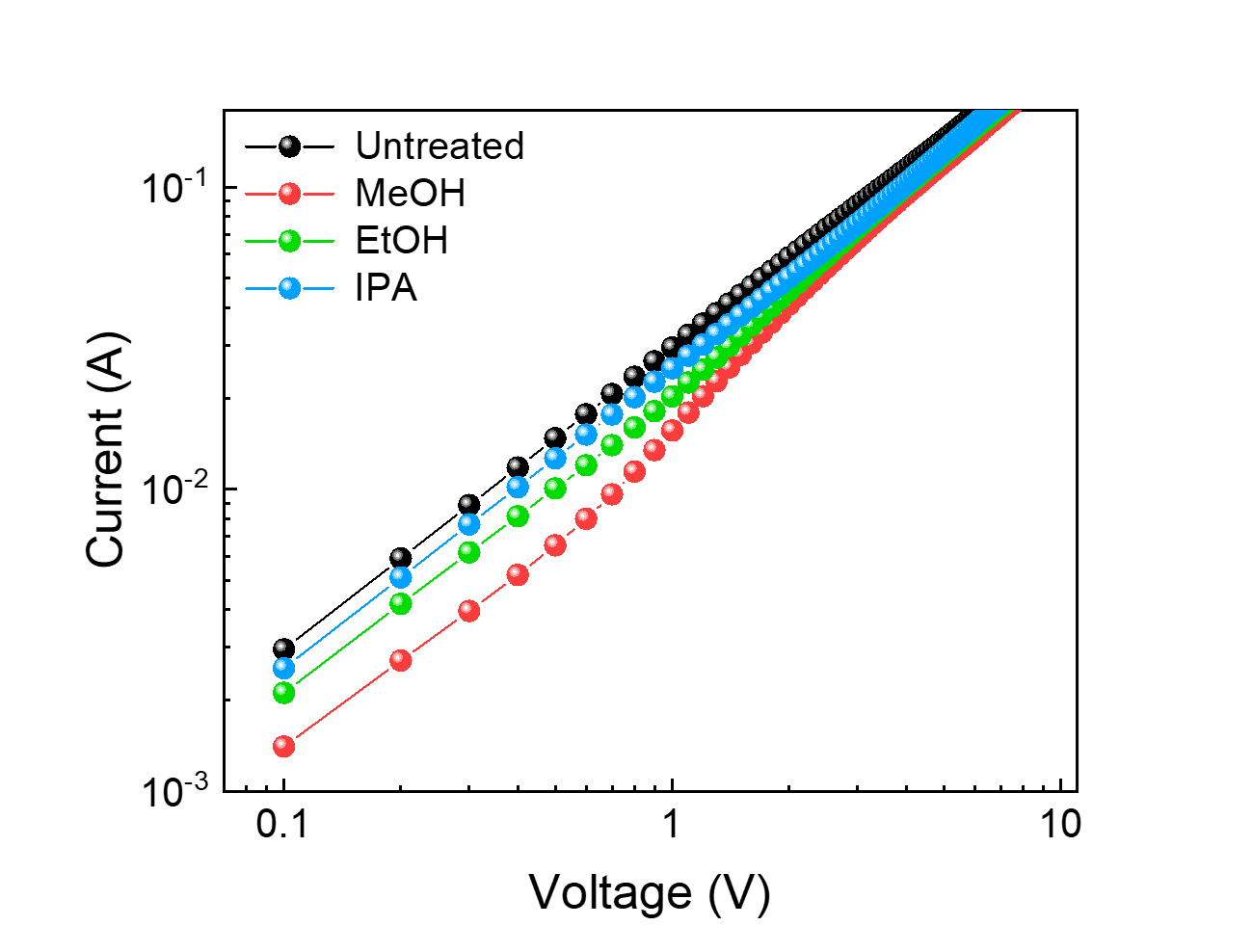


**Fig. S4.** Current‒voltage characteristics of the EODs (UT-ZMO and MeOH-, EtOH-, and IPA-treated ZMOs) under different conditions.


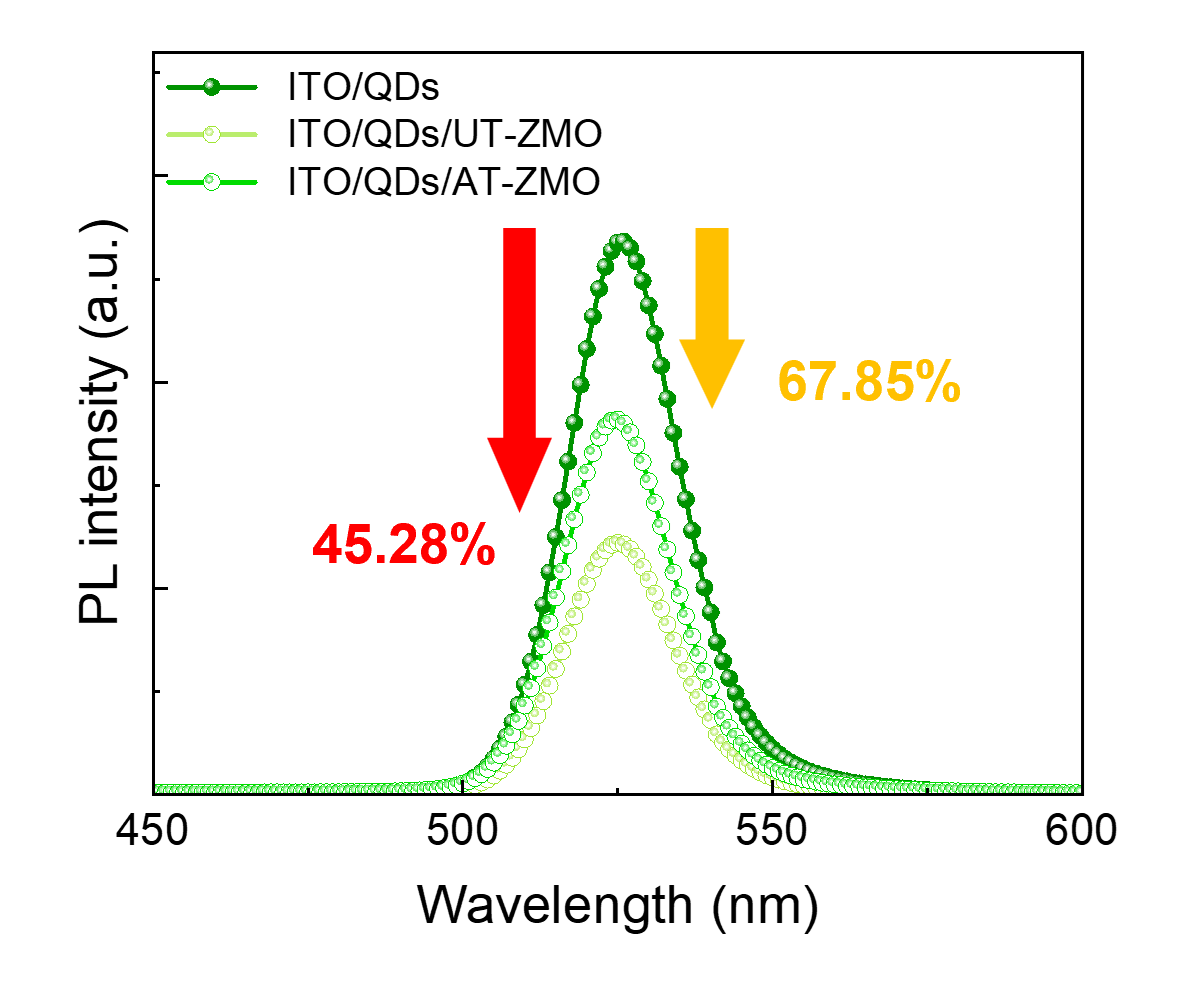


**Fig. S5.** Variations in the PL intensity of the CdZnSeS/ZnS QD films containing UT-ZMO and MeOH-treated ZMO.


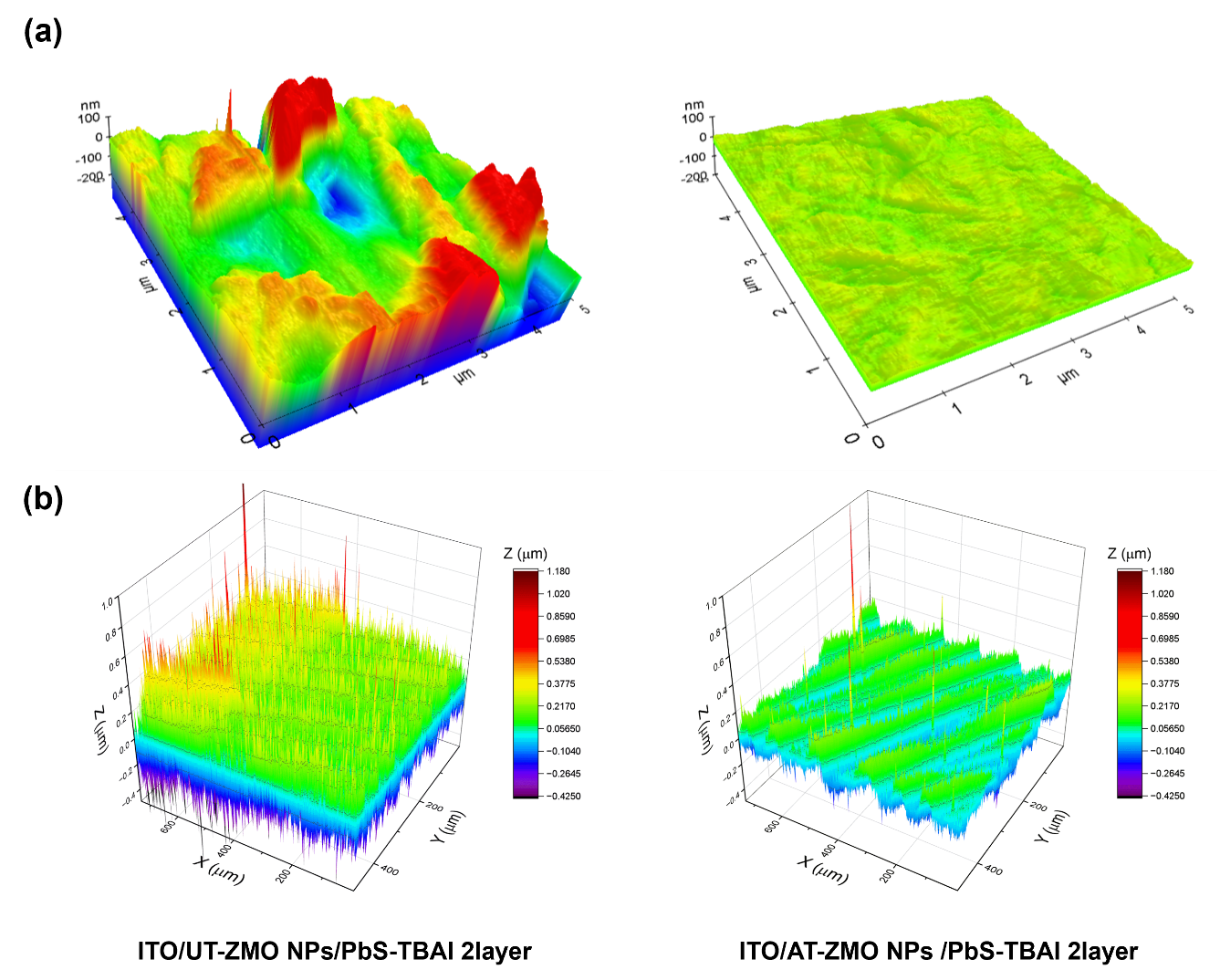


**Fig. S6.** AFM and optical profiler images of the ITO/ZMO NPs/PbS-TBAI structure under different conditions.


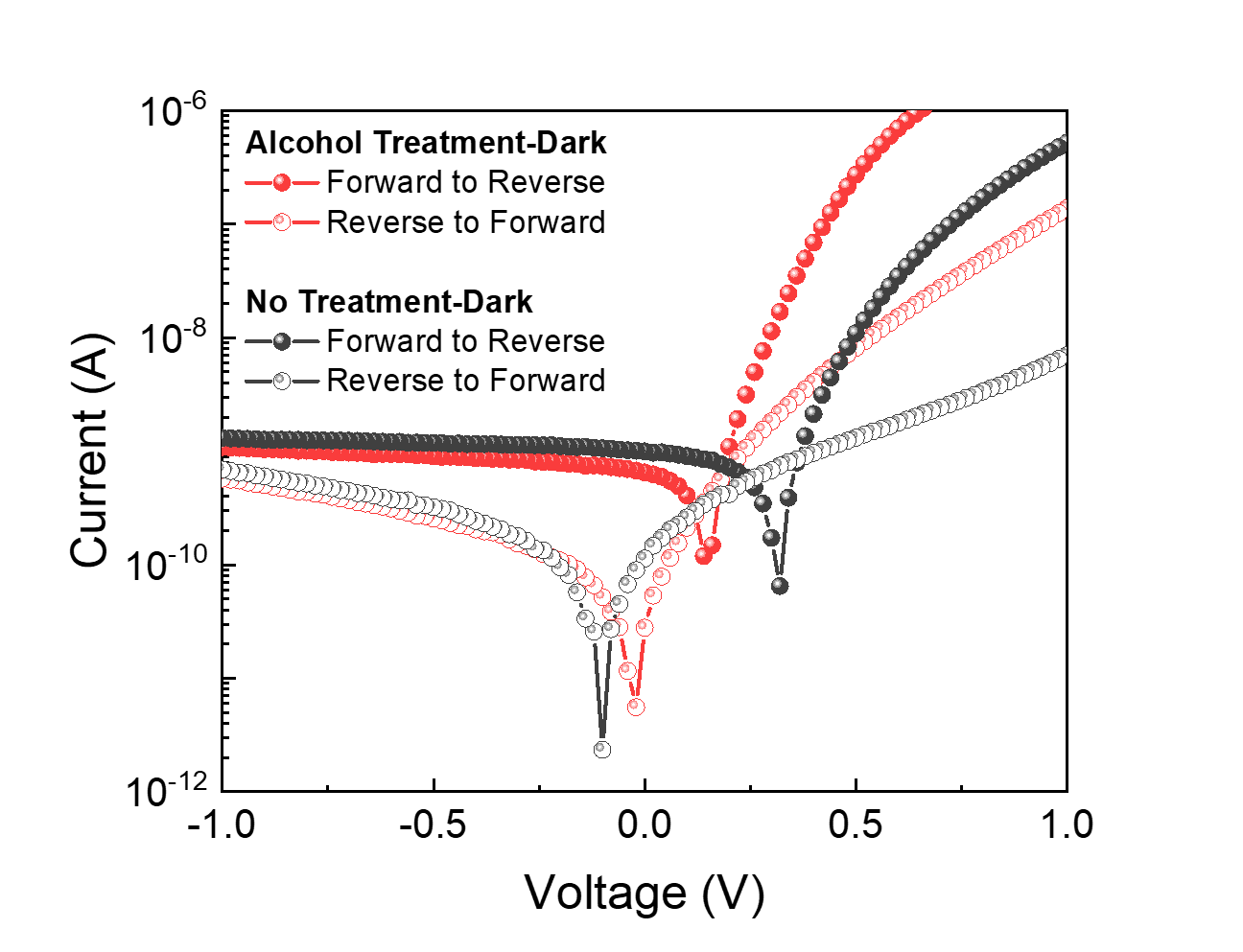


**Fig. S7.** Dark-state I–V characteristics of UT-ZMO and AT-ZMO photodiodes measured under forward-to-reverse and reverse-to-forward voltage sweeps


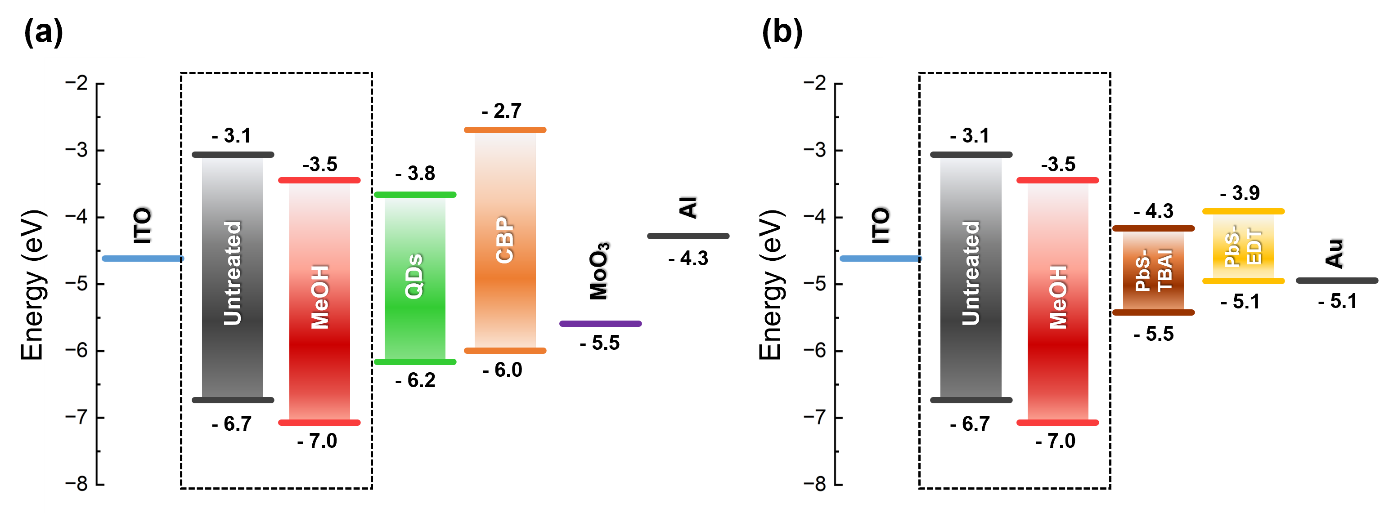


**Fig. S8.** Energy band levels of the QLEDs and QPDs containing UT-ZMO and MeOH-treated ZMO NP ETLs.

**Reference**

[1] K. Lee, J. lee, Y. Bae, H. Roh, W.H. Jung, J. Lim, J. Kim, J. Roh, Interfacial Modification of ZnO/ZnMgO Bilayer for Efficient and Stable InP Quantum Dot Light-Emitting Diodes via Ultraviolet Ozone Treatment, *ACS Appl. Mater. Interfaces.* 16, 55658-55665 (2024). <https://doi.org/10.1021/acsami.4c09900>.

[2] S.Y. Yoon, Y.j. lee, H. Yang, D.Y. Jo, H.M. Kim, Y. Kim, S.M. Park, S. Park, H. Yang, Performance Enhancement of InP Quantum Dot Light-Emitting Diodes via a Surface-Functionalized ZnMgO Electron Transport Layer, *ACS Energy Lett.* 7, 2247–2255 (2022). <https://doi.org/10.1021/acsenergylett.2c01065>.

[3] Y. Park, M. lee, H. Seo, D. Shin, D. Hahm, W.K. Bae, J. Kim, J. Kwak, Efficient and stable InP quantum-dot light-emitting diodes formed by premixing 2-hydroxyethyl methacrylate into ZnMgO, *J. Mater. Chem. C.* 12, 7270–7277 (2024). <https://doi.org/10.1039/d4tc00837e>.

[4] D. Heo, J. H. Chang, D. Shin, J. Kwak, W. Bae, H. Lee, Modified Zinc Magnesium Oxide for Optimal Charge-Injection Balance in InP Quantum Dot Light-Emitting Diodes, *Adv. Opt. Mater.* 11, 2202256 (2024). <https://doi.org/10.1002/adom.202202256>.
